# Designing a Positive Health Dialogue Tool for Adolescents and Young Adults: A Mixed Methods Study

**DOI:** 10.1111/hex.70042

**Published:** 2024-10-24

**Authors:** Marja van Vliet, Machteld Huber, Sigrid van der Zanden

**Affiliations:** ^1^ Institute for Positive Health Utrecht the Netherlands; ^2^ Public Health Service Brabant Zuid‐Oost Eindhoven the Netherlands

**Keywords:** adolescents, health promotion, integrated care, positive Health, shared decision making, young adults

## Abstract

**Objectives:**

Being able to express and address strengths and needs related to health is an important asset to enhance resilience during emerging adulthood. Towards this end, we aimed to develop a specific version of the My Positive Health (MPH) dialogue tool for this developmental period (ages 16–25). By adopting a broad perspective on health and striving for applicability in various settings, this tool ultimately seeks to promote integrated collaboration across various domains (healthcare, social care, school).

**Methods:**

The tool was co‐designed with end users, using mixed methods: a descriptive cross‐sectional survey questionnaire (*N* = 118) followed by qualitative focus groups and interview sessions (*N* = 36). Participants were Dutch citizens (mean age 21.6; 79% female) who were stratified by educational level and chronic disease status (yes/no). The final selection of the content of the tool was made during expert sessions.

**Results:**

A preference appeared for the following dimensions to be part of the dialogue tool: *My body, My feelings and thoughts, Meaningfulness, Quality of life, Participation* and *Daily life*. Each dimension was operationalised by aspects (43 in total) to facilitate reflection and dialogue. The following new aspects that are typical for emerging adulthood were formulated and included in the dialogue tool: *‘*Confidence in yourself*’*, ‘Being in control’, *‘*Having confidence in the future*’*, *‘*Self‐expression*’*, ‘Meaningful relationships*’*, ‘Being able to work or study*’* and ‘Ability to plan*’*. Other aspects derived from the adult and children's versions of the MPH dialogue tool.

**Conclusion:**

A dialogue tool was designed for individuals aged 16–25 in various health‐ and vulnerability‐related conditions and with applicability in various domains.

**Patient or Public Contribution:**

The development of the dialogue tool was specifically driven by the needs expressed by the users themselves. Planned public and patient contribution comprised consultation and collaboration in (i) design, (ii) recruitment, (iii) focus group sessions, (iv) analysis and discussion of the data and (v) dissemination.

**Trial Registration:** Not applicable.

## 1 Introduction

The age period roughly between 16 and 25 years can be referred to as ‘emerging adulthood’. During this challenging period, specific developmental tasks are acquired, such as taking care of one's own living situation and finances, building close relationships, attaining educational goals and transitioning from the education phase to a working career [1]. Evidence shows that health problems in adolescence are related to a variety of adverse life outcomes, including poor employment conditions and social exclusion [2, 3]. This period of ‘emerging adulthood’ is even more challenging for adolescents with chronic conditions compared with their healthy peers, as they face additional challenges to achieve developmental milestones [4]. The group of adolescents with chronic conditions is growing, as more and more paediatric patients reach adulthood due to improved treatment of life‐threatening diseases, over recent decades [5, 6, 7]. Therefore, it is important to address aspects of experienced functioning and situational factors, both preventive and as part of the shared decision‐making process between patient and healthcare provider or social worker [8]. Paying attention to these aspects may lead to a lower healthcare dropout rate, positive (future) psychosocial functioning, motivation, optimism and illness cognition [7, 9, 10]. However, interventions in various settings to support optimal development towards adulthood seem to be scarce and those available mainly focus on specific patient groups [8, 11, 12].

**Figure 1:**
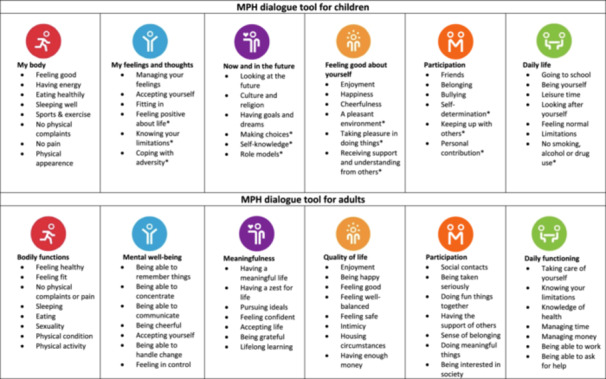
Overview of dimensions and aspects of the My Positive Health dialogue tool for children and adults. *demarcated questions for the 12–16‐year age group. Images reproduced with permission by the Institute for Positive Health, the Netherlands, 2024.

The literature shows that focusing on individual situations and experiences and acquiring a grip on their assets in life are key ingredients for successful interventions for vulnerable adolescents and young adults [13, 14]. Additionally, research indicates that insight into one's own perceived health status is a prerequisite for self‐management skills and healthier behaviour [15, 16]. The dynamic concept of health by Huber et al. [17], which describes health as ‘the ability to adapt and self‐manage in the face of social, physical and emotional challenges’ aligns with these principles. For the Dutch setting, this dynamic concept was further operationalised into Positive Health (PH), which reflects a broad perception of health and emphasises the ability to overcome challenges and lead a meaningful life. The focus is not on limitations and imperfections, but on possibilities and health resources [18]. The concept has evolved into a leading framework for Dutch healthcare policy, encouraging self‐management, shared decision‐making and integrated collaboration [19, 20, 21]. To facilitate this in practice, the My Positive Health (MPH) dialogue tool for adults was developed [22]. This dialogue tool aims to provide individuals with insight into their own health and stimulate self‐reflection, which may ultimately foster resilience. Resilience can be described as ‘the capacity of individuals to navigate their way to the psychological, social, cultural, and physical resources that sustain their well‐being, and their individual and collective capacity to negotiate the resources to be provided in culturally meaningful ways’ [23, p. 225]. By completing 44 statements distributed over six dimensions (bodily functions, mental well‐being, meaningfulness, quality of life, participation and daily functioning), mean scores for each of the dimensions are graphically represented in a spider web. This presents a subjective overview of someone's life status, which can then be used during consultations with, for example, healthcare professionals and social workers to discuss the person's perceived health and to direct attention to those areas that are relevant to them. In this light, someone's most essential needs, desires and abilities are discussed. The next step is to determine whether the person involved has a desire to change, and, if so, in which of the dimensions. The final step is to identify whether they would need support in making the required changes. In this way, the tool helps connect to personal motivation and creates insight into their health resource to foster resilience [24]. The tool is digitally offered as an adult version (https://vragenlijsten.mijnpositievegezondheid.nl/adults), a children's version (focus on ages 8–16; https://vragenlijsten.mijnpositievegezondheid.nl/child) and a simple version (for adults with reading comprehension problems). The digital tools are widely used in the Netherlands, with more than 350,000 unique users (in May 2024) since their introduction in 2016 and 2017, respectively. In addition, paper versions of the MPH dialogue tools are used across a wide range of care centres (in Dutch, English, German, Spanish, Icelandic and Japanese). See Figure 1, for an overview of dimensions and aspects of the adult and children's versions of the MPH dialogue tool. To ensure that the dimensions and aspects of the dialogue tools are in line with their target populations (i.e., adults and children), content and terminology were derived from responses by patients and citizens to the question about what they considered to be indicators of ‘health’ [8, 18].

Research indicates that integrating the MPH decision‐making tool in primary care for adults not only enhances overall well‐being and quality of care from the patient's perspective but also contributes to increased job satisfaction among healthcare professionals [25, 26, 27, 28]. Furthermore, providing a common language seems to cultivate collaboration between the social and health domains [29]. In a primary care experiment, the implementation of the MPH tool resulted in a noteworthy 25% decrease in hospital referrals and an overall reduction in healthcare expenditure. Patient preferences, as identified by the MPH tool, encompass a wide range of dimensions, prompting the need for a comprehensive offering from both the healthcare and social domains, facilitating enhanced collaboration between these two sectors and more cost‐effective care [24, 28, 30].

Considering the distinct age period between 18 and 25 years, a specific version of the MPH dialogue tool that takes the challenges of emerging adulthood into account seems a valuable addition. This need was also expressed by a panel of young adults with chronic diseases (JongPIT). The specific version was designed for adolescents and young adults with and without a chronic disease as well as professionals, to promote self‐reflection among individuals by enhancing their insight into their own health, well‐being, strengths and needs, thus ultimately fostering overall resilience. Simultaneously, it aims to enhance understanding among healthcare professionals and social workers about their patients' experiences. When needed, they can collaborate to identify and choose the support preferred by their patients, all with the overarching aim of assisting individuals in building a meaningful life. Therefore, the objective of this study was to develop a version of the MPH dialogue tool specifically for the period of emerging adulthood that could be used in various settings, based on the adult and children's versions.

## 2 Material & Methods

### 2.1 Design

For this study, we applied a design‐based research approach. Iterative cycles of analysis, design, development, testing and refinement were conducted via collaboration between researchers and end users [31]. Design of the study was developed by the first author in collaboration with members of JongPIT and consisted of two phases. During the first phase, data were collected among Dutch participants by using a mixed methods design—a descriptive cross‐sectional survey followed by qualitative focus group sessions and interviews. Results from the first phase were refined in the second phase, in expert sessions.

Planned public and patient contributions comprised consultation and collaboration in (i) design, (ii) recruitment, (iii) focus group sessions, (iv) analysis and discussion of the data and (v) dissemination.

## 3 Methods

### 3.1 Phase 1

#### 3.1.1 Participants and Data Collection

In this study, we employed a mix of purposeful and convenience sampling methods. Eligible individuals were reached through networks associated with institutions (e.g., schools) collaborating with iPH and a network of adolescents and young adults with chronic diseases (via JongPIT). People were invited to participate in both the survey and focus group sessions/interviews. In cases of time constraints or other personal circumstances preventing them from physical participation, they could opt to only fill out the survey. Participants in the survey and focus group sessions/interviews were stratified over six groups: absence or presence of a chronic illness and education level (lower, moderate, higher). Within each of these stratified groups, participants were of all ages between 18 and 25 years, both sexes and from various cultural backgrounds. One focus group session was held per stratified group. For practical reasons, five additional interviews were held with those on limited time. Collection of quantitative and qualitative data took place in the Autumn 2020.

Because the tool was initially designed for young adults aged 18–25, participants within this age range were targeted for the survey and focus group sessions. However, after it was decided that this age group should be expanded, we also included 16‐ and 17‐year‐olds. This added nine participants to the survey bringing the total to 118 and 3 to the focus group to a total of 36 participants. See Table 1, for an overview of the number of participants in the survey, focus group sessions and interviews.

**Table 1:** Number of participants in the quantitative and qualitative study.

Chronic illness	Level of education^a^	Focus group/interviews	Number of participants in focus groups/interviews	Number of respondents in quantitative study
Yes	Higher	Focus group	5	29
	Moderate	Interviews and focus group	4	8
	Lower	Interviews	4	6
No	Higher	Focus group	8	42
	Moderate	Focus group	7	23
	Lower	Focus group	8	10
Total			36	118

a Higher = BSc, MSc, PhD; moderate = intermediate vocational education (In Dutch: MBO level 4); lower = below level 4, vocational education.

#### 3.1.2 The Survey

The online survey had three sections. It started with questions regarding demographics and other characteristics, such as age, sex, education level, cultural background and chronic illnesses. Then, following an introduction about PH and the dialogue tool, two questions were posed per dimension: first, respondents were asked if, for the dimension, they preferred the term from the adult or children's version or would prefer another term. For the second question, a list of all aspects of the adult and children's versions was provided for each corresponding dimension (Figure 1). Respondents were asked to select those that they considered most important, with a maximum of seven aspects per dimension, but there was also room to add aspects that respondents felt were missing. Before distributing the survey, it was tried out on three young adults from existing contacts. Participants were invited by email to complete the online survey.

#### 3.1.3 Focus Group Sessions and Interviews

Focus group sessions and interviews were structured as follows. First, an introduction to PH and the corresponding tool was provided. Next, the group results from the survey (containing answers from each focus group) were shown per dimension and, subsequently, discussed. This included the dimensions and aspects that were chosen the most and the least. Participants were encouraged to indicate their preferences and were asked if they were missing any aspects. The focus group sessions and interviews were conducted in a setting chosen by the participants (e.g., at home, school or university).

Focus groups were chaired by the first author and assisted by the third author, and individual interviews were conducted by the third author. Participants were not known to the interviewers beforehand. The meetings were audio recorded, transcribed, summarised and presented to the participants for approval.

#### 3.1.4 Phase 2

##### 3.1.4.1 Expert Sessions

Experts were selected in consultation with the JongPIT panel, after which sessions were organised to propose the initial choice of dimensions and aspects. The sessions included a group discussion with seven young adult experience experts, an interview with an independent senior researcher specialising in young adults with chronic illnesses, and an interview with the architect of the new dynamic concept of health and the MPH dialogue tools for adults and children.

### 3.2 Data Analysis

#### 3.2.1 Phase 1

##### 3.2.1.1 Quantitative Analysis

Descriptive statistics were used to summarise the demographic characteristics of the participants and their survey responses. Multiple logistic regression was used for associations between the respondents' characteristics and their preferences regarding the terms used for the main dimensions (significance level *p* < 0.05). Data were analysed using SPSS 24.0 (IBM Corp, 2016).

##### 3.2.1.2 Qualitative Analysis

Manifest content analysis was used to analyse the data [32]. According to this procedure, meaning units (i.e., constellations of words or statements that relate to the same central meaning) were identified and systematically coded, based on what was literally said (i.e. the manifest content). Category terms were formulated based on the existing dimensions and aspects of the adult and children's versions of the dialogue tool. Meaning units were sorted into these categories and, for new aspects, additional category terms were formulated. This step was initially conducted by the first and third authors and results were subsequently discussed with the whole study team including representatives of JongPIT. This process resulted in an initial choice of terms for the dimensions and corresponding aspects. The qualitative software programme NVivo version 8 [33] was used to guide the analysis.

#### 3.2.2 Phase 2

##### 3.2.2.1 Expert Sessions

All experts, independently from each other, considered the proposed dimensions and corresponding aspects and discussed the issues.

The final step involved a consensus meeting involving all authors. Differences of opinion were discussed, selected aspects were agreed on by consensus and subsequently clustered into main categories (the dimensions) and related aspects. During each step, the authors all had access to the transcripts. Subsequent outcomes and decisions from each analysis step served as an audit trail to guide the discussions.

### 3.3 Ethical Considerations

According to the Dutch Social Support Act (WMO), this study was exempt from ethical approval. Before participation in the survey, focus group sessions and interviews, all participants provided their informed consent after having received information about the background and purpose, mode of participation and confidentiality. Participation was entirely voluntary. All data were handled confidentially.

## 4 Results

### 4.1 Descriptives

Table 1 shows the number of participants. In total, 118 respondents completed the survey, 36 of which participated in the focus groups and interviews.

Table 2 shows overall socio‐demographic characteristics.

**Table 2:** Socio‐demographic characteristics of the study population.

	Chronic disease (*N* = 43)	No chronic disease (*N* = 75)	Total (*N* = 118)
Mean age (SD)	21.9 (2.9)	21.4 (2.7)	21.6 (2.8)
Sex (%)			
Male	17	26	21
Female	83	74	79
Educational level			
Higher	67	56	60
Moderate	19	31	26
Lower	14	13	14
Work/study (%)^a^			
Study	60	76	70
Work	40	45	43
Trainee	14	23	20
None/other	17	1	7
Living situation(%)			
Parental home	48	54	52
Student home	12	22	18
Independent (individual/with partner)	40	24	30
Cultural background (%)			
Dutch	90	89	90
Turkish/Moroccan	2	3	3
Surinamese/Antillian	—	4	3
Other	7	4	5
Mean physical health (SD)^b^	5.5 (2.0)	7.2 (1.4)	6.6 (1.8)
Mean mental health (SD)^b^	6.0 (2.1)	6.9 (1.6)	6.6 (1.8)

a Multiple answers per respondent possible.

b Self‐reported health status on a Likert scale from 1 to 10.

### 4.2 Main Dimensions

#### 4.2.1 Quantitative Results

Table 3 shows the percentages for the preferred terms per dimension, as indicated by the survey respondents. The results show the variation in preferences among the stratified groups.

**Table 3:** Preferred terminology based on the dimensions.

*N* = 118^a^						
Chronic disease	Yes			No		
Educational level	Lower	Moderate	Higher	Lower	Moderate	Higher
Dimension 1						
Bodily functions (%)	33	75	24	50	76	40
My body (%)	67	13	66	50	24	58
Other/don't know (%)	—	13	10	—	—	3
Dimension 2						
Mental well‐being (%)	17	38	31	60	44	38
My feelings and thoughts (%)	50	50	48	20	35	36
Other/don't know (%)	33	13	21	20	22	26
Dimension 3						
Meaningfulness (%)	17	63	17	40	22	43
Now and in the future (%)	33	25	59	40	44	36
Other/don't know (%)	50	13	24	20	35	22
Dimension 4						
Quality of life (%)	33	63	67	30	35	57
Feeling good about yourself (%)	33	25	17	50	39	29
Other/don't know (%)	33	13	16	20	26	14
Dimension 5						
Participation (%)	67	88	79	80	70	79
Other/don't know (%)	33	13	21	20	30	21
Dimension 6						
Daily functioning (%)	17	38	41	50	44	38
Daily life (%)	50	50	40	30	30	48
Other/don't know (%)	33	13	4	20	26	14

^a^Percentages may exceed 100% due to rounding.

Multiple logistic regression showed that the various terms preferred (in the adult and children's versions) for each dimension were not significantly associated with age, gender or illness. For level of education, significant associations were found for dimensions 1 and 4, but not for the other dimensions. Respondents with a lower or higher educational level were respectively 4.71 and 6.70 times more likely to prefer ‘My body*’* over ‘Bodily functions’, compared with those with a moderate level of education (lower educational level: OR = 4.71; CI: [1.24, 17.97]; higher educational level: OR = 6.70; CI: [2.38, 18.87]. Furthermore, respondents with a higher educational level were significantly more likely to prefer ‘Quality of life*’* over ‘Feeling good about yourself’, compared with those with a lower and moderate level of education (OR = 2.53; CI: [1.07, 6.99]).

#### 4.2.2 Qualitative Results

During the focus group meetings and interviews, participants indicated their preference for the terms ‘My body’ and ‘My feelings and thoughts’ for the first and second dimension, respectively, as those terms appeared more personal to them, thereby inviting individuals to think about their experienced physical and mental functioning.

No distinct preference was indicated for the term for the third dimension. Some participants regarded ‘Now and in the future’ as a clear and practical term, while others favoured ‘Meaningfulness’.

With respect to the fourth dimension, participants preferred the term ‘Quality of life’, as they considered this to be more comprehensive than ‘Feeling good about yourself’. The participants agreed on the term ‘Participation’ for the fifth dimension —which is used in both the adult and the children's version of the tool—which they considered appropriate. Regarding the sixth dimension, the term ‘Daily life’ was preferred over ‘Daily functioning’, although the scores on both terms were close together.

The results from the expert participants in the survey and focus group sessions showed their consensus on the terms ‘My body’, ‘My feelings and thoughts’, ‘Quality of life’, ‘Participation’ and ‘Daily life’. For the third dimension, they preferred ‘Meaningfulness’ over *‘*Now and in the future’, as they considered that this term more fully captured the aspects of personal fulfilment in life, which is more significant for this age group.

#### 4.2.3 Corresponding Aspects Per Dimension

##### 4.2.3.1 Quantitative Results

Table 4 provides an overview of the most selected aspects per dimension. It shows that the respondents with and without a chronic disease mostly chose similar aspects.

**Table 4:** Overview of aspects per dimension considered most important by respondents in the survey.

My body				My feelings and thoughts				Meaningfulness			
**Chronic disease**		**No chronic disease**		**Chronic disease**		**No chronic disease**		**Chronic disease**		**No chronic disease**	
**Aspect**	**%**	**Aspect**	**%**	**Aspect**	**%**	**Aspect**	**%**	**Aspect**	**%**	**Aspect**	**%**
No physical complaints	74	No physical complaints	59	Accepting yourself	72	Accepting yourself	67	Looking at the future	67	Having goals and dreams	55
Having energy	60	Having energy	57	Feeling positive about life	60	Feeling positive about life	57	Having goals and dreams	67	Having a zest for life	47
Sleeping	56	Physical condition	53	Knowing your limitations	58	Managing your feelings	53	Making choices^a^	60	Being grateful^a^	47
No pain^a^	47	Feeling healthy	53	Being able to concentrate	51	Being able to concentrate^a^	49	Acceptance	60	Lifelong learning	45
Physical condition	47	Fitness^a^	48	Managing your feelings	47	Coping with adversity^a^	49	Lifelong learning	53	Looking to the future	45
Feeling healthy	44	Sleeping	48	Being able to handle change^a^	44	Being able to communicate	49	Feeling confident	51	Feeling confident	44
Sports & exercise	44	Sports & exercise	44	Being able to remember things^a^	42	Knowing your limitations	39	Having a zest for life	49	Acceptance	43

**Quality of life**				**Participation**				**Daily life**			
**Chronic disease**		**No chronic disease**		**Chronic disease**		**No chronic disease**		**Chronic disease**		**No chronic disease**	
**Aspect**	**%**	**Aspect**	**%**	**Aspect**	**%**	**Aspect**	**%**	**Aspect**	**%**	**Aspect**	**%**
Being happy	70	Feeling safe	61	Social contacts	72	Social contacts	69	Taking care of yourself	70	Taking care of yourself	69
Enjoyment	63	Enjoyment	59	Being taken seriously	67	Being taken seriously	60	Being able to ask for help^a^	49	Knowing your limitations	56
Taking pleasure in doing things	53	Being happy	57	Receiving support and understanding from others	65	Receiving support and understanding from others	55	Knowing your limitations	63	Managing money	55
Feeling safe	53	Taking pleasure in doing things	48	Sense of belonging	49	Doing fun things together	44	Being able to work	44	Managing time	48
Balance	51	Having enough money	47	Personal contribution^a^	47	Sense of belonging	43	Managing time	44	Being able to work	40
Receiving support and understanding from others a	49	Feeling well‐balanced	39	Self‐determination^a^	44	Friends*	41	Managing money	40	Being yourself	39
A pleasant environment^a^	40	Feeling good a	39	Doing fun things together	44	Being interested in society^a^	39	Being yourself	40	Knowledge of health	37
Having enough money	40							Knowledge of health	40		

a Aspects selected by only one group (with/without a chronic disease).

##### 4.2.3.2 Qualitative Results

###### 4.2.3.2.1 Dimension 1

Participants expressed their preference for the aspects of *‘*no physical complaints’, ‘feeling healthy’, ‘having energy’, ‘sleeping*’, ‘*fitness’ and ‘sports & exercise’. They emphasised the importance of being pain‐free, having sufficient energy, and the benefits of good sleep for maintaining fitness and overall well‐being. Furthermore, participants indicated that ‘eating healthily’ should also be included as an aspect of bodily functions. The aspect of ‘no pain’ was considered relevant by the participants, as they shared the opinion that pain has a large impact on their experienced health and daily life. Furthermore, they indicated that ‘physical appearance’ is important during adolescence and young adulthood. Some respondents added that this aspect becomes even more important for those with a visible medical condition.

###### 4.2.3.2.2 Dimension 2

Participants expressed their preference for the aspects of ‘accepting yourself*’, ‘*feeling positive about life*’, ‘*concentration’ and *‘*managing your feelings*’.* They highlighted the importance of self‐acceptance, maintaining a positive outlook, concentration and effective emotional management. They also preferred ‘dealing with change’ over ‘coping with adversity’, as they found this to be a more positive term. They indicated that the period of adolescence and young adulthood is marked by changes with respect to studying, housing and work. Dealing with these changes was therefore regarded as an important skill. Furthermore, according to participants, *‘*being in control’ was considered important, as this period of life is often rather demanding, and feeling healthy calls for having control. They also valued the aspect of ‘being able to communicate’ but had some doubts about the terminology, noting that the ability to communicate involves a certain amount of reciprocity. It was therefore suggested to name the aspect ‘self‐expression’ and to include it in dimension 4 (‘Participation’). ‘Knowing your limitations’ was considered important as well, but participants considered this to be part of ‘Daily life’.

###### 4.2.3.2.3 Dimension 3

Participants expressed their preference for the aspects of *‘*having goals and dreams*’, ‘*looking at the future*’, ‘*having a zest for life’ and ‘making choices*’.* They particularly highlighted that thinking about and preparing for the future is crucial for their sense of direction and motivation. Participants also agreed that ‘having a meaningful life’ is important, as doing things that are considered valuable is of major importance in a person's life. There was some confusion about the meaning of the term ‘feeling confident’. According to the participants, it was unclear whether this meant having confidence in oneself or being confident about life. Therefore, they suggested changing it to ‘having confidence in the future’. There was similar uncertainty about ‘acceptance’, which was therefore suggested to be changed to ‘accepting situations’. Participants disagreed about the importance of ‘gratitude’; those who had experienced more profound life events appeared to value this aspect more than participants with relatively less life experience.

###### 4.2.3.2.4 Dimension 4

Participants expressed their preference for the terms *‘*happiness*’, ‘*enjoyment*’, ‘*feeling safe*’, ‘*a pleasant environment’ and *‘*making ends meet’*.* They highlighted the importance of having a good life despite certain limitations, being accepted by one's environment and having the financial means to engage in activities that add value to life. Participants also indicated that feeling well‐balanced was often a challenge to them, in the face of their demanding lives. However, according to them the term ‘balance’ was too vague. Therefore, they suggested an elaboration to ‘experiencing balance’ Furthermore, participants indicated the value of having a pleasant place to live, referring to the physical housing circumstances and with whom you live. They therefore agreed on the term ‘a pleasant environment’, since this includes both social and physical elements.

###### 4.2.3.2.5 Dimension 5

Participants expressed their preference for the aspects of *‘*social contacts*’, ‘*being taken seriously*’, ‘*doing fun things with other people*’, ‘*belonging’ and *‘*receiving support and understanding from others*’.* They highlighted the negative consequences of not being taken seriously and feeling excluded, noting the detrimental effect on their well‐being. Some participants indicated that ‘social contacts’ could also refer to more superficial relationships with, for example, classmates or colleagues. However, they regarded the term ‘friends’ rather childish and informal. Although participants were of the opinion that building close relationships was an important element of this stage of life, there was disagreement about a fitting term. Terms like sexuality or intimate relationships made many participants feel uncomfortable. Therefore, a number of participants suggested the term ‘meaningful relationships’. This could include close relationships with friends, sexual partners and family members.

###### 4.2.3.2.6 Dimension 6

Participants expressed their preference for the aspects of ‘taking care of yourself’, ‘knowing your limitations’, ‘being able to ask for help’ *and* ‘knowledge about health’, particularly highlighting the significance of being independent and setting boundaries, especially for young adults with chronic conditions striving for equality with their peers. Participants confirmed the importance of work for leading a meaningful life. However, as this stage of life may involve study and/or work, the term for this aspect was combined to ‘being able to work or study’, ‘Managing time’ and *'*managing money’ were both considered important by the participants. As both are related to the ability to plan, they were combined to form the new aspect ‘ability to plan’. Although ‘no smoking, alcohol or drug use’ was considered less important among respondents to the survey, they did indicate the impact of these addictive substances on daily life and believed it would be important to discuss openly, for instance, during a health consultation. Therefore, they suggested to include this aspect in the dialogue tool.

### 4.3 Expert Session

During the expert sessions, the following issues were discussed: As the aspects of ‘pain’ and ‘complaints’ were both considered important during the focus group sessions, these were combined into one aspect: ‘no physical complaints or pain*’.*

Despite the focus group disagreeing on the importance of the aspect of ‘gratitude’, the experts considered it an important aspect that is often developed during adolescence/young adulthood. They therefore included it as an aspect of the third dimension.

Although participants in the focus groups considered ‘housing and living environment’ less important, the experts selected regarded *housing quality* as important for the quality of life and health status, and this aspect can reveal a homeless situation.

Experts and respondents in the focus groups recommended that the 18–25 age range be expanded to 16–25 years. Experts confirmed the notion of respondents in the focus group sessions and interviews that the transition from adolescence into adulthood often starts before the age of 18. Another argument was related to the transfer from paediatric to adult care. Experts indicated that it would be beneficial for young adults to start using the tool in the period before having to transfer to adult care, to prepare themselves for gaining self‐awareness and self‐management skills, which are required in adult care.

**Figure 2:**
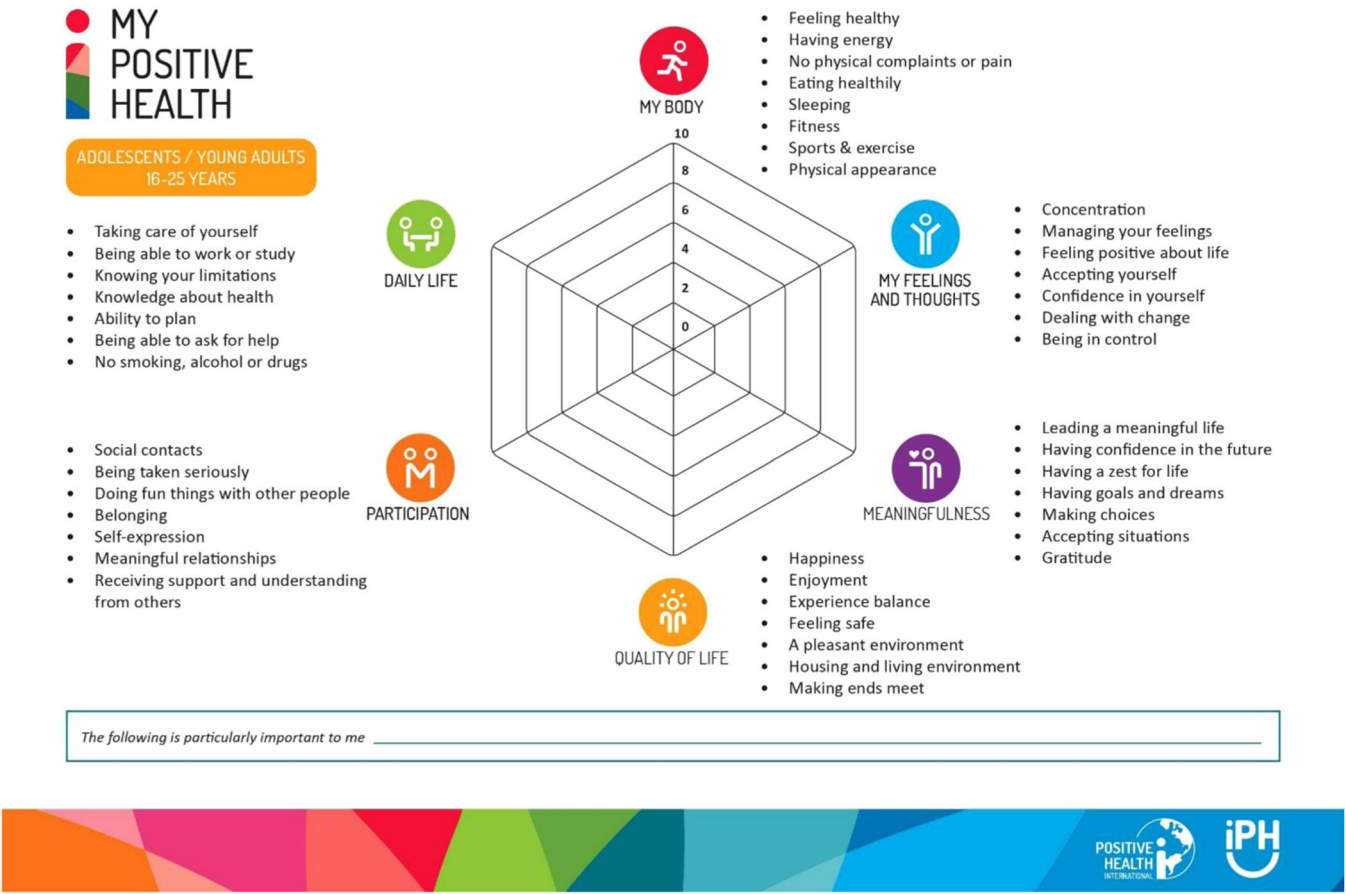
My Positive Health dialogue tool for adolescents and young adults. Reproduced with permission from the Institute for Positive Health and Positive Health International, the Netherlands, 2024.

Figure 2 provides an overview of the final selection of dimensions and aspects. Overall, the dialogue tool includes 43 aspects; 16 of which are derived from the adult version, 11 from the children's version and 7 are used in both versions. In addition, seven new aspects were added and two were slightly renamed.

## 5 Discussion

In this study, we developed a new version of the MPH dialogue tool, specifically for adolescents and young adults aged between 16 and 25 and related healthcare professionals. The tool for adolescents and young adults consists of six dimensions *(My body, My feelings and thoughts, Meaningfulness, Quality of life, Participation* and *Daily life*). Each of which contains seven aspects, except for *My body*, which contains eight. The aspects are a mixture of those in the adult and the children's versions. Seven new aspects were added, namely ‘Confidence in yourself’, ‘Being in control’, ‘Having confidence in the future*’*, *‘*Self‐expression*’*, ‘Meaningful relationships*’*, ‘Being able to work or study*’* and ‘Ability to plan’. Multiple logistic regression analyses show that the preference for children's or adult terms to describe the dimensions was associated with level of education, rather than age, gender or having a chronic disease. Young adults with lower and higher educational levels were both more likely to choose the children's term (‘My body*’)* with respect to the first dimension. Furthermore, in the fourth dimension, respondents with a higher educational level significantly more often preferred the adult term ‘Quality of life’, compared with those with a lower or moderate education level.

The MPH dialogue tool for adolescents and young adults aims to increase resilience and self‐management skills by providing users with insight into their own health, thus stimulating self‐expression. Furthermore, it is aimed at increasing the responsivity of professionals (in several domains) to genuinely increase the strengths and meet the needs of adolescents and young adults. Thus, people's insight into their perceived health is regarded as an individual asset, stimulating an active approach towards their health‐related situation. The focus is on active engagement, which is regarded as an important asset for building resilience [34, 35]. The tool offers a wide array of health‐related aspects, including those typical for the phase of emerging adulthood [1]. Reflection on the results from the spiderweb helps individuals focus attention on those areas that are relevant and of interest to them. Moreover, the focus is on preferred future directions instead of on things that went wrong in the past. This will increase intrinsic motivation, which is indicated as an important factor that also contributes to resilience [35].

Instead of targeting specific groups (e.g., young adults with or without disease, or specific vulnerable groups) or settings (e.g., schools, hospitals), the tool is designed for young adults in general. This aligns well with the thought that, due to the multidimensional nature of resilience, interventions that cut across a variety of behaviours are more effective than focusing on specific problem areas [34]. The study's findings support this approach, showing relatively small differences between preferred dimensions and aspects per subgroup. However, differences in term preferences between higher and lower educated young adults suggest that additional attention should be paid to appropriate guidance that takes educational level into account. This is in line with previous research indicating that this attention should be directed to persons less skilled in self‐managing their own health [36]. When discussing the MPH dialogue tool, the primary focus is often on the assets of the individual. However, resilience is not solely derived from internal factors but rather is the outcome of a synergy between internal and external assets. Therefore, it is crucial to extend the attention beyond individual resources and also consider environmental factors that contribute to fostering overall health [24].

One of the main arguments for lowering the age range to 16–25 years is related to the transfer from paediatric to adult care. The transfer to adolescent care is known to be challenging due to differences between paediatric and adult care and the changing interests of young adults [37]. In the transition period, there is a high dropout rate from health care among adolescents with chronic diseases [10, 38]. Inadequate self‐management is associated with higher dropout rates [39]. To prevent people from opting out of hospital care, which could lead to a deterioration of their chronic condition, it is essential to learn self‐management skills. This includes taking responsibility for one's own health and acquire autonomy and health‐ and disease‐related knowledge [40]. The MPH dialogue tool contributes to this process by stimulating self‐awareness of one's perceived health and preferences. By starting to use the tool before the age of 18, users can become acquainted with it, which will contribute to an easier shift from paediatric to adult care. At the same time, the age range of the children's version is adapted from 8 to 18 and 8 to 16. In general, we suggest that the 16–25 age range is not set in stone, but depends on the developmental stage of the user.

### 5.1 Methodological Considerations

One of the strong points of this study, which was stratified by educational level, is the relatively high number of individuals involved. This enabled us to include a varied population from a wide range of backgrounds, which allowed us to gain insight into the differences in opinions between groups. Another strong point is the process of co‐design. The active involvement of the end users during the development process ensures that the tool will suit their preferences [31, 41]. The main limitation of the study is the sample size for the quantitative results. In addition, we complemented purposeful sampling with the uncontrolled method of snowball sampling. Therefore, the quantitative results should be cautiously interpreted and cannot be generalised to the whole population. However, as the selection of aspects was largely based on the qualitative interviews, this has supposedly had a minimal effect on the tool's selected dimensions and aspects.

The content of the MPH tool for adolescents and young adults is based solely on the preferences of end users and experts, with no information available on its psychometric properties, including construct validity. In a previous study, the six‐factor structure of the dialogue tool for adults was found to be valid. However, while suitable for dialogue, it was not effective as a measurement tool for scientific purposes. A factor analysis extracted 17 aspects from the original 42, which can complement the dialogue tool for measurement purposes. We recommend performing a factor analysis on this tool for adolescents and young adults to assess its psychometric properties and determine if a valid measurement set of items can be extracted.

Since its introduction, the dialogue tool for adolescents and young adults has been implemented in a digital environment (https://vragenlijsten.mijnpositievegezondheid.nl/junior) and made available in a paper version. The JongPIT panel facilitated further dissemination by organising an event and sharing the results and tools within their network. Several schools and healthcare providers have started to use the MPH dialogue tool. Initial studies suggest the potential of the adult tool to promote health‐related behaviour among various populations [31, 42]. Such evaluations of the MPH tool for young adults are needed to gain more insight into its effects. Furthermore, more research is required to increase the understanding of its underlying mechanisms and its impact on interprofessional collaboration.

In conclusion, the MPH dialogue tool for adolescents and young adults is designed to be used by individuals aged 16–25 with a variety of health conditions and vulnerabilities. Its applicability extends to various settings, including healthcare practices, social work environments and schools. The tool is aimed to contribute to the overall well‐being of individuals during emerging adulthood and promote the emergence of more responsive professionals. The ultimate goal is to empower individuals to navigate developmental milestones with greater ease, enabling them to build a more meaningful life.

## Author Contributions

**Marja van Vliet:** Conceptualisation, writing–original draft, methodology, supervision, formal analysis, project administration, investigation, software, data curation, funding acquisition. **Machteld Huber:** Funding acquisition, writing–review & editing, validation, supervision, investigation, conceptualisation, resources, visualisation. **Sigrid van der Zanden:** Conceptualisation, investigation, writing–review & editing, formal analysis, methodology, software.

## Consent

Before participation in both the survey and focus groups/interviews, all participants provided their informed consent after having received information about the background and purpose, mode of participation and confidentiality. Participation was entirely voluntary and data were handled confidentially.

## Conflicts of Interest

The authors declare no conflict of interest.

## Data Availability

Reasonable requests for sharing data can be made by sending an email to the corresponding author.
